# r*Citrus*BBC: a bacterial resource to mine for new agricultural probiotics for citrus

**DOI:** 10.1128/MRA.00408-23

**Published:** 2023-09-29

**Authors:** Lucía Solaz-Rodenas, Ramón Penyalver

**Affiliations:** 1 Centre for Plant Protection and Biotechnology of the Valencian Institute of Agricultural Research (IVIA), Moncada, València, Spain; University of Delaware College of Engineering, Newark, Delaware, USA

**Keywords:** citrus microbiome, rhizospheric bacteria, beneficial bacteria, agricultural probiotics

## Abstract

A culture collection of 147 selected bacteria from the rhizospheric citrus microbiome is available at the Valencian Institute of Agricultural Research (València, Spain). The data include information on plant growth-promoting (PGP) traits published to date and the presence of PGP-related genes in the available genomes of the different bacterial species.

## ANNOUNCEMENT

Mutualistic microbes present in root-associated communities provide a potential source of plant growth-promoting rhizobacteria (PGPR) (1). Plant probiotics have enormous economic potential and are accepted in sustainable agriculture (2, 3). Unraveling plant bacterial microbiomes can help to select potentially host-beneficial microbes (4). As a resource that can be used to mine for new agricultural probiotics for citrus, we present a *r*hizospheric *Citrus B*eneficial *B*acterial *C*ollection (r*Citrus*BBC) of 147 potentially host-beneficial microbes encompassing 17 genera and belonging to 51 bacterial species.

Bacterial citrus microbiomes were previously described using uncultured and cultured approaches, and the untapped diversity of host-associated bacteria served as a source of benefits (5). In this sense, bacterial citrus microbiomes were surveyed to select potentially host-beneficial microbes (4, 5). Briefly, a prevalent “core-citrus” bacterial microbiome was selected by picking 544 operational taxonomic units (OTUs) shared among samples within and across two *Citrus* rootstock genotypes grown in the same soil for more than 20 years. In parallel, a large number of bacteria were isolated from the same rhizospheres by a culturomic approach applying seven culture conditions (5). Four hundred eighty-two representative isolates were identified by double-stranded sequencing of the 16S rRNA gene. For inclusion in this collection, a given 16S rRNA sequence of each isolate was compared with the reference 16S rRNA sequence of the selected core-citrus OTUs assigned to the same genus. A total of 147 isolates were closely associated (>98% similarity) with 25 OTUs, thus generating a collection of rhizospheric microbes potentially beneficial for citrus (4, 5).

Plant growth-promoting (PGP) traits in each bacterial species already published to date were surveyed by a Web Of Science databases-indexing as indicated in Table 1. Most of the bacterial species present in the r*Citrus*BBC collection announced herein were already described as PGP bacteria for other crops (Table 1). Conversely, genome mining analyses show the presence of PGP-related genes in most of the bacterial species present in the r*Citrus*BBC collection (Table 1). Moreover, despite the limited number of commercialized bacterial bioproducts used in agriculture, our collection of bacteria is dominated by genera that are already commercially available as plant probiotics or have been tested experimentally on other crops, such as *Arthrobacter*, *Pseudomonas*, *Bacillus*, *Rhizobium*/*Agrobacterium*, *Erwinia*/*Pantoea*, and *Flavobacterium* (3, 6, 7). Together, all these data show the potential of the r*Citrus*BBC collection announced herein as a source to mine for new agricultural probiotics for citrus crops after their selection through specific future *in planta* research projects.

**TABLE 1:** Most of the bacterial species present in the r*Citrus*BBC collection announced herein are already described as plant growth-promoting rhizobacteria (PGPR) for other crops and contain PGP-associated genes

		Bibliographic search^*b*^			Genome mining^*e*^					
Bacterial species	No. of isolates in r*Citrus*BBC collection^*a*^	PGP activity	PGP activities^*c*^	Specific PGP traits^*d*^	Genome availability in ortholog databases^*f*^	AcdS^*g*^	IaaM	IaaH	PhoA	PqqC
*Bacillus wiedmannii*	22	Yes	1 2 (salt, drought, and heavy metals) 3 (fungal diseases) 4	1 2 [indole-3-acetic-acid (IAA)] 3 4	Yes***	Yes**	Yes**	Yes**	Yes***	Yes***
*Agrobacterium tumefaciens*	11	Yes	1 2 (salt and arsenic) 4	1 2 (IAA) 3 5	Yes*	No*	No*	Yes*	No*	No*
*Pseudomonas putida*	10	Yes	1 2 (flooding, salt, and drought) 3 (various soil-borne pathogens)	1 2 (IAA) 5	Yes**	No**	Yes*	Yes**	No**	No**
*Pseudomonas jessenii*	9	Yes	1 2 (flooding, salt, cold, heavy metals, and drought) 3 (phytopathogens) 4	1 2 (IAA) 4 5	Yes**	No**	Yes*	Yes**	No**	No**
*Arthrobacter globiformis*	6	Yes	1 2 (iron, salt) 4	2 (IAA) 3 4 5	Yes*	Yes*	No*	Yes*	No*	No*
*Microbacterium trichothecenolyticum*	6	Yes	1 2 (mercurium)	2 (IAA) 5	Yes**	Yes**	Yes*	Yes**	Yes*	No**
*Pseudomonas oryzihabitans*	6	Yes	1 2 (salt) 4	1 2 (IAA, indole-3-lactic acid and indole-3-carboxylic acid) 3 4 5	n.a.					
*Paracoccus litorisediminis*	5	No			Yes***	Yes**	Yes***	Yes**	Yes***	No***
*Stenotrophomonas maltophilia*	5	Yes	1 2 (salt) 3 (fungal pathogen) 4	1 2 (IAA and gibberellic acid) 3 4 5	Yes***	Yes**	Yes***	Yes**	Yes***	No***
*Pseudomonas taiwanensis*	4	Yes	1 3 4	2 (IAA) 4 5	Yes*	No*	Yes*	No*	Yes*	No*
*Pseudoxanthomonas japonensis*	4	Yes	1 3 (nematode)		Yes*	No*	Yes*	No*	Yes*	No*
*Pseudomonas helmanticensis*	4	Yes	1 2 (salt, drought, heavy metals) 4	1 2 (IAA) 4 5	n.a.					
*Pseudomonas silesiensis*	3	Yes	1 2 (cold) 3 (fungal and bacterial pathogens) 4	1 2 (IAA) 4 5	Yes*	No*	Yes*	No*	Yes*	No*
*Rhodococcus koreensis*	3	No			Yes*	No*	Yes*	No*	Yes*	No*
*Rhodococcus wratislaviensis*	3	No			Yes***	Yes**	Yes*	Yes*	Yes***	Yes**
*Pseudomonas baetica*	2	Yes	1 2 (salt, drought) 4	1 2 (IAA) 4 5	Yes*	Yes*	No*	No*	No*	No*
*Pseudomonas entomophila*	2	Yes	1 2 (drought) 3 (antifungal activity) 4	1 2 (IAA and gibberellic acid) 3 4 5	Yes***	Yes**	Yes*	Yes*	Yes***	Yes***
*Pseudomonas frederiksbergensis*	2	Yes	1 2 (salt, heavy metals) 4	2 (IAA) 4 5 6	Yes**	Yes*	No*	No*	Yes*	No*
*Pseudomonas lini*	2	Yes	1 2 (drought) 3 (antifungal) 4	1 2 (IAA) 3 4 5	Yes***	No***	Yes**	Yes*	Yes***	No***
*Nocardia globerula*	2	Yes	3 (antifungal)		n.a.					
*Bacillus mobilis*	2	No			Yes*	Yes*	Yes*	Yes*	No**	No**
*Microbacterium yannicii*	2	No			Yes**	No**	Yes*	Yes**	Yes**	No**
*Erwinia billingiae*	2	Yes	3 (antifungal)		Yes**	No**	Yes*	Yes**	No**	No**
*Microbacterium profundi*	2	No			Yes**	No**	Yes*	Yes**	No**	No**
*Pseudomonas plecoglossicida*	2	Yes	1 2 (salt and aluminum) 3 (antifungal) 4	1 2 (IAA) 4 5	n.a.					
*Pseudomonas brassicacearum*	1	Yes	1 2 (salt) 3 4	1 2 (IAA) 3 5	Yes**	Yes*	Yes*	Yes**	No**	No**
*Pseudomonas monteilii*	1	Yes	1 2 (drought) 4	2 (IAA, gibberellic acid, and cytokines) 4 5	Yes**	Yes**	No**	Yes*	Yes**	Yes**
*Pseudomonas japonica*	1	Yes	1 2 (salt)	5	Yes*	No*	No*	No*	No*	Yes*
*Flavobacterium frigidimaris*	1	No			Yes***	Yes***	Yes*	Yes*	Yes***	No***
*Kribbella swartbergensis*	1	No			Yes*	Yes*	No*	No*	Yes*	No*
*Rhizobium herbae*	1	No			Yes*	Yes*	Yes*	No*	No*	Yes*
*Rhizobium giardinii*	1	Yes	4	3	Yes***	Yes***	Yes***	Yes***	No***	Yes***
*Bacillus cereus*	1	Yes	1 2 (salt, metals) 4	1 2 (IAA) 3 4 5	Yes***	Yes***	Yes***	Yes**	No***	Yes***
*Bacillus mojavensis*	1	Yes	1 3 (nematode)		Yes***	Yes***	Yes***	Yes***	No***	Yes***
*Bacillus paramycoides*	1	Yes	1 2 (salt, drought, metals) 4	1 2 (IAA) 4 5	n.a.					
*Bacillus toyonensis*	1	Yes	1 2 (salt, metals) 3 (antifungal)	2 (IAA, cytokinin, and nonindole phenylacetic acid) 4	Yes**	Yes**	Yes**	Yes**	No**	Yes**
*Microbacterium paraoxydans*	1	Yes	1 2 (metals) 3 (antifungal)	1 2 (IAA) 5	Yes*	Yes*	Yes*	Yes*	No*	Yes*
*Pedobacter steynii*	1	No			n.a.					
*Pedobacter westerhofensis*	1	No			Yes***	Yes***	Yes***	Yes**	No***	Yes***
*Janthinobacterium lividum*	1	Yes	2 (cold) 3 (antifungal)		Yes***	Yes***	Yes**	Yes**	No***	Yes***
*Terribacillus goriensis*	1	No			Yes***	Yes***	Yes***	Yes**	No***	Yes***
*Arthrobacter cupressi*	1	No			Yes***	Yes***	Yes***	Yes***	No***	Yes***
*Arthrobacter humicola*	1	Yes	1 2 (cold) 3 (antifungal) 4	2 (IAA, indole-3-butyric acid, gibberellin, zeatin, and abscisic acid) 3 4	Yes***	Yes***	Yes***	Yes**	No***	Yes***
*Arthrobacter luteolus*	1	Yes	1 2 (salt)		n.a.					
*Arthrobacter pascens*	1	Yes	1 2 (salt) 3 4	4 5	Yes*	No*	No*	No*	Yes*	No*
*Pantoea rodasii*	1	Yes	1 2 (salt) 4	2 (IAA) 4 5	Yes**	No**	Yes*	Yes*	No**	No**
*Bacillus litoralis*	1	Yes	1	2 (IAA) 5	n.a.					
*Erwinia endophytica*	1	Yes	2 (salt)		Yes**	Yes*	Yes*	Yes*	No**	Yes**
*Erwinia tasmaniensis*	1	Yes	4	2 (IAA) 3 4	Yes*	No*	No*	Yes*	No*	Yes*
*Agromyces fucosus*	1	No			Yes***	No***	Yes*	Yes**	Yes**	No***
*Nocardia salmonicida*	1	No			Yes**	No**	Yes*	Yes*	Yes**	No**

^*a*^ The r*Citrus*BBC #ID of isolates is indicated in Penyalver et al. 2022. https://doi.org/10.1016/j.nbt.2022.06.002.

^*b*^ For the bibliographic search for PGP activity, the terms (*bacterial species* + PGP* OR *plant*) were used for indexing the Web Of Science database.

^*c*^ PGP activities are indicated by numbers: 1, PGP; 2, tolerance to abiotic stress; 3, tolerance to biotic stress and 4, plant nutrition.

^*d*^ Specific PGP traits are also indicated by numbers: 1, acc deaminase; 2, production of phytohormones; 3, nitrogen fixation; 4, phosphate solubilization; 5, siderophore production; and 6, potassium solubilization.

^*e*^ The presence of PGP-related genes in each bacterial species of the collection was achieved by mining the corresponding genes in available genomes using three ortholog databases: eggNOG 6.0 (www.eggnog6.embl.de/); OrthoDB (www.orthodb.org) and KEGG (www.genome.jp/kegg/).

^*f*^ The availability of bacterial species genomes is indicated by Yes followed by *, **, or *** depending on the number of databases where the corresponding genome is available. n.a. indicates bacterial species that are not available in any databases.

^*g*^ The presence of PGP-related genes in each bacterial species of the collection is indicated by Yes or No followed by *, **, or *** depending on the number of databases in which the gene was or was not identified.

The collection is available as frozen strains preserved in PBS buffer containing 50% glycerol, maintained at −80°C in duplicate, and deposited in two separated research buildings on the IVIA premises in València, Spain (http://hdl.handle.net/20.500.11939/8245).

## Data Availability

The r*Citrus*BBC #ID of isolates is indicated in Penyalver et al. (5). 16S rRNA gene sequences from each isolate are deposited at NCBI under accession numbers OK298500 to OK298946. To acquire isolates or have questions, please contact Ramón Penyalver at penalver_ram@gva.es.
